# A laparoscopic vascular blocking forceps used for renal carcinoma combined with tumor thrombus

**DOI:** 10.1590/S1677-5538.IBJU.2020.0063

**Published:** 2020-12-20

**Authors:** Xinfei Li, Guangpu Ding, Xuesong Li, Liqun Zhou

**Affiliations:** 1 Peking University First Hospital Institute of Urology Department of Urology Beijing China Department of Urology, Peking University First Hospital. Institute of Urology, Peking University National Urological Cancer Center, Beijing, China.

## Abstract

**Objectives:**

To discuss the feasibility and efficacy of a laparoscopic vessel blocking forceps in laparoscopic inferior vena cava (IVC) thrombectomy (1–3).

**Materials and methods:**

The patient was secured in a left lateral decubitus position. The surgical field was built with 4-trocar. The laparoscopic vessel blocking forceps was used to block the IVC partially. With the help of the forceps, we completed a retroperitoneal laparoscopic radical nephrectomy and IVC thrombectomy.

**Results:**

The patient was a 73-year-old female. The tumor was located on the right side. Based on the preoperative radiology examination, the tumor thrombus extended from the right renal vein into the IVC, and the cephalic extent of tumor thrombus was 1.6cm above the renal vein. The preoperative stage was T3b, and the Mayo grade of the tumor thrombus was grade I. The operation was successfully completed without conversion to open surgery. The operation time was 159 minutes, and the estimated blood loss was about 50ml. No blood transfusion was needed. The postoperative hospital stay was 10 days. No operation related complication was observed. Postoperative pathology showed diffusely poor differentiated carcinoma, and the pathological stage was T3bN0.

**Conclusion:**

The laparoscopic vascular blocking forceps can clamp vessels without damaging the vessels. Vascular blocking forceps is suitable for laparoscopic surgical field. We recommend such a vascular blocking forceps for laparoscopic thrombectomy in patients with renal carcinoma and Mayo grade 0-I tumor thrombus. It may be used to clamp other blood vessels temporarily or control bleeding during laparoscopy in the future.
